# A Case Report of Dramatically Increased Thyroglobulin after Lymph Node Biopsy in Thyroid Carcinoma after Total Thyroidectomy and Radioiodine

**DOI:** 10.1155/2016/6471081

**Published:** 2016-02-29

**Authors:** Mandana Moosavi, Stuart Kreisman

**Affiliations:** ^1^Vancouver Coastal Health, Canada; ^2^Division of Endocrinology, St. Paul's Hospital, 1081 Burrard Street, Vancouver, BC, Canada V6Z 1Y6

## Abstract

Thyroglobulin (Tg) is an important modality for monitoring patients with thyroid cancers, especially after thyroidectomy followed by radioiodine (RAI). It is also used as a marker for burden of thyroid tissue whether malignant or benign. Although there have been several reports of rising serum Tg transiently after thyroid biopsy in intact glands and following palpation or trauma, there are no reports in the literature of elevation in Tg after biopsy of suspicious lesions in thyroidectomized patients. In this paper we report a fascinating case of a considerable and initially worrying, although ultimately transient, rise in Tg in a patient 2 years after total thyroidectomy and RAI ablation after fine needle aspiration (FNA) of a suspicious thyroid bed nodule that was proven positive.

## 1. Background

Tg is a storage form for thyroxine and triiodothyronine. It is synthesized only by follicular thyroid cells. Due to its cellular specificity, Tg is an excellent marker that has long been used for surveillance after thyroidectomy and after RAI ablation in thyroid cancer patients. The American Thyroid Association Guidelines recommend routine measurement of Tg along with neck ultrasounds as the principal modalities of patient follow-up [1]. If Tg levels are elevated, ultrasound followed by biopsy of growing or suspicious nodules is recommended; however there are no guidelines on timing of biopsy with respect to Tg measurements [1]. Tg is known to increase with benign thyroid swelling, after trauma and surgery as well as with FNA of the intact thyroid gland itself [1]. It is thus potentially important to know when to check serum Tg and how to properly interpret the result as it can cause significant concern for the physician and the patient, suggesting not only cancer recurrence, but also disease acceleration.

There have been several reports of Tg rising after FNA of nodules and normalizing within a few days. The half-life of circulating Tg is 65 hours [2]. In one study, 12 patients with a solitary cold nodule or multinodular goitre had serum Tg measured before and 5–60 minutes after FNA. Seven of them had statistically significant increases in serum thyroglobulin ranging from 35.4 ng/mL to 58 ng/mL with an increase of 305% being seen in one patient with follicular carcinoma [2]. This study, although in a small sample, suggested a greater rise in Tg in malignant nodules. In another study, an increase in Tg ranging from 35% to 341% was also seen 5 minutes to 3 hours after FNA irrespective of whether the biopsied nodule was benign or malignant. The Tg values normalized 2 weeks after FNA in all the subjects. There was no change in thyroid-stimulating hormone, total thyroxine, free thyroxine, or free triiodothyronine with FNA [3]. A similar rise of 35–77% in Tg was also seen in 22 out of 25 patients measured before and 60 minutes after FNA in a third study where the Tg rise persisted for up to 15 days [4]. There have not been any reports of a rise in serum Tg or Tg antibody in patients who have been thyroidectomized with or without radioiodine and are undergoing biopsies for possible recurrence. In this paper we aim to discuss a case of Tg rise after biopsy in a patient after thyroidectomy and RAI.

## 2. Patient

We report a case of a patient who had increase in Tg after biopsy for assessment of a suspicious ultrasound finding after thyroidectomy and radioiodine for thyroid cancer. She is a 46-year-old woman originally from Ukraine who was exposed to the Chernobyl disaster. Her family history was negative for thyroid cancer. On an ultrasound done for follow-up of her multinodular goiter, there was a suspicious nodule of 7 mm, with biopsy consistent with papillary thyroid carcinoma. Left thyroid lobectomy showed that her tumor had positive margins with no lymphatic or vascular invasion. She underwent completion thyroidectomy 8 months later, which showed two additional foci of 2 and 1 mm of papillary carcinoma in the right lobe, with clear margins. Histology report revealed conventional papillary carcinoma with enlarged nuclei. She initially did not have any RAI therapy, but her Tg started to rise without stimulation to 6 and 8 from previous values of 1 for both cases (see Table 1) and a subsequent biopsy of a left thyroid bed nodule returned positive. Modified neck exploration was performed and histology showed metastases in 4 of 12 lymph nodes. She was then treated with 100 mCi of radioiodine by thyroid hormone withdrawal (her TSH was 111 with Tg of 6 ng/mL at the time). The 7-day posttreatment scan showed some activity in the thyroid bed but no evidence of distant metastases. Part of the reason she was investigated so intensely was due to her extreme anxiety and concern about her prognosis, despite reassurance. One year after her third surgery (the neck exploration), ultrasound in the left thyroid bed showed two hypoechoic nodules of 10 mm and 4 mm. Biopsy of both nodules was again positive for papillary thyroid carcinoma. One month before biopsy her TSH was 11.2 and Tg was 0.9 ng/mL, at which point her thyroxine dose was increased. Another Tg was ordered around the time of biopsy and by chance was done after biopsy by the patient. To our surprise her thyroglobulin increased to 39 ng/mL, 3 days after biopsy (time zero in Table 1). Given patient's already anxious mindset, this finding led to even more anguish. Because of this and a hypothesis that the rise may have been biopsy related, Tg was repeated six days later and had already fallen to 10.3 ng/mL and rapidly came back down to 0.9 ng/mL six weeks after (see Table 1). Her second neck exploration occurred 3 weeks after second biopsy that also showed four nodes positive for papillary thyroid carcinoma. She then had 150 mCi MBq of RAI followed by a whole body scan a week later, which showed neither residual activity in the thyroid bed nor evidence of functioning thyroid metastases. In our hospital, the surgical approach in patients with lymph node metastasis involves total thyroidectomy with central compartment lymphadenectomy and modified neck dissection. Her last two thyroid ultrasounds were after second lymph node excision, both of which did not demonstrate any suspicious findings. One year after her second neck exploration, for the first time she had undetectable Tg and Tg antibody. Her most recent Tg was <0.1 ng/mL and TSH was 0.03 two years after biopsy.

## 3. Discussion

American Thyroid Association Guidelines recommend any suspicious nodules be investigated with ultrasound and possible biopsy, while routine measurement of Tg is not part of the standard work-up of nodules prior to cancer diagnosis. It is known that Tg can be elevated postsurgically as well as in association with benign and malignant thyroid tissue. However, although there have been several reports of rising Tg after FNA, there have not yet been any reports of Tg rise after FNA in thyroidectomized patients regardless of radioiodine or lymph node status. In our case, Tg transiently rose to 39 ng/mL and at the time there was nothing in literature to help interpret this finding. Although this was hypothesized to be biopsy related, it could also have represented an ominous marker of accelerating disease, leading to much anxiety for the patient. Interassay variation can be significant, often due to variation in antithyroglobulin antibodies used, or the heterogeneity of Tg itself due to alternative processing. With intra-assay variation, small fluctuations can be expected in the same patient [5], but not such high values as in our case.

It is important to know the significance of findings on both ultrasound and biopsy as well as values of Tg and anti-Tg antibody. As reported in the case above, an increase in Tg can cause extreme concern for both the patient and the physician; thus it is important to both know when to check Tg values and interpret them in the appropriate clinical context in order to avoid unnecessary investigations. Current guidelines do not address the timing of Tg with respect to biopsy, and we had previously never given any instructions to our patients in this regard. In general, unless stimulated, Tg rise in recurrent thyroid cancer is slow and steady and the rapid rise in this case led to concern of the disease somehow having become more aggressive. In this case biopsy effect was hypothesized as being the more likely explanation from the outset and confirmed when Tg dropped shortly after. Hence it is our recommendation that future guidelines should instruct on timing Tg measurement before any planned biopsy. On the other hand, if interpreted carefully thyroglobulin after biopsy may have some diagnostic utility. While the lack of any rise in a nondiagnostic biopsy would be reassuring, presumably biopsy of benign residual after surgical thyroid bed tissue would also lead to some rise in Tg. Whether the magnitude of such rise differs from that following biopsy of malignant lesions could be worth investigating.

## Figures and Tables

**Table 1:** Summary of thyroglobulin levels and thyroglobulin antibodies in patient discussed (day zero is biopsy date). Tg: thyroglobulin in ng/mL; Tg-Ab: thyroglobulin antibody.

	After total thyroidectomy: 4 yr	After total thyroidectomy: 3 yr	After total thyroidectomy: 3 yr	After total thyroidectomy: 3 yr	After neck dissection: 1 yr	After RAIl: 30 days	Day 3	Day 6	Day 42	Day 365	Day 730
Tg (ng/mL)	1	6	7	8	6 (stimulated Tg with TSH 111)	0.9 (one year after RA ablation)	39	10.3	0.9	<0.1	<0.1
Tg-Ab	<10	<10	<10	<10	<10	<10	<10	<10	<10	<10	<10

## Abbreviations

Tg: Thyroglobulin
FNA: Fine needle aspiration
RAI: Radioiodine.
